# Controlling the corrosion and cathodic activation of magnesium via microalloying additions of Ge

**DOI:** 10.1038/srep28747

**Published:** 2016-06-28

**Authors:** R. L. Liu, M. F. Hurley, A. Kvryan, G. Williams, J. R. Scully, N. Birbilis

**Affiliations:** 1 Department of Materials Science and Engineering, Monash University, Clayton, VIC, Australia; 2 College of Engineering, Department of Materials Science and Engineering, Boise State University, Boise, Idaho, USA; 3 Materials Research Centre, College of Engineering, Swansea University, Singleton Park, Swansea, Wales, UK; 4 Department of Materials Science and Engineering, The University of Virginia, Charlottesville, VA, USA

## Abstract

The evolution of corrosion morphology and kinetics for magnesium (Mg) have been demonstrated to be influenced by cathodic activation, which implies that the rate of the cathodic partial reaction is enhanced as a result of anodic dissolution. This phenomenon was recently demonstrated to be moderated by the use of arsenic (As) alloying as a poison for the cathodic reaction, leading to significantly improved corrosion resistance. The pursuit of alternatives to toxic As is important as a means to imparting a technologically safe and effective corrosion control method for Mg (and its alloys). In this work, Mg was microalloyed with germanium (Ge), with the aim of improving corrosion resistance by retarding cathodic activation. Based on a combined analysis herein, we report that Ge is potent in supressing the cathodic hydrogen evolution reaction (reduction of water) upon Mg, improving corrosion resistance. With the addition of Ge, cathodic activation of Mg subject to cyclic polarisation was also hindered, with beneficial implications for future Mg electrodes.

Magnesium (Mg) alloys are the lightest of the structural engineering metals and therefore offer several potential advantages with respect to specific strengths^1^,^2^. In recent decades, Mg alloys have been increasingly sought for use as structural materials to improve energy efficiency and meet light weighting demands^1^,^2^,^3^,^4^,^5^, whilst with the advent of portable electronics, and the prospect of Mg as use as an electrode material, even greater focus has turned to the durability of Mg, namely its corrosion and electrochemical properties^6^,^7^,^8^.

The well documented and high rates of corrosion for Mg and its alloys remains a barrier to the wider application of Mg-based systems^1^. In aqueous environments, Mg is thermodynamically unstable^9^,^10^ and highly electroactive. As such, the corresponding corrosion potentials for Mg in aqueous environments are well below −1 V_SHE_^11^ assuring that the reduction of water (2H_2_O + 2e^−^ 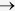 H_2_ + 2OH^−^) is the principal cathodic reaction that accompanies anodic dissolution (Mg 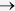 Mg^2+^+ 2e^−^)^12^.

Mg corrosion occurs readily in most aqueous and atmospheric environments, as Mg does not possess a passive surface film for pH < 11^10^,^13^. Furthermore, during open circuit exposure in aqueous solution, copious amounts of hydrogen are generated as a result of the cathodic reaction balancing rapid anodic dissolution^14^. Moreover, when Mg is anodically polarised, the rate of the partial cathodic reaction, namely the hydrogen evolution reaction (HER), increases with applied anodic potential^15^. This phenomenon, often termed the “negative difference effect”^16^, is indicative of (i) a persistent cathodic reaction^17^ and (ii) a cathodic reaction that is catalysed by anodic dissolution^15^. As such, the corrosion and dissolution of Mg is notionally under ‘cathodic control’. Conversely, the anodic kinetics of Mg dissolution present a low Tafel slope^18^, consistent with Mg being weakly polarisable. This means that the variations in the kinetics of the cathodic reaction upon Mg (where the cathodic reaction has a relatively high Tafel slope and is polarisable) have a significant influence on the corrosion and electrochemistry of Mg.

Based on the above introduction of Mg electrochemistry in aqueous solutions, the ability to retard the cathodic kinetics upon Mg, would offer a ‘kinetic’ form of corrosion control. Further, the ability to retard the cathodic activation of Mg (cathodic activation being the terminology used to describe the increase in cathodic kinetics as a result of anodic dissolution), is therefore one tenable approach for the kinetic restriction of Mg corrosion. To this end however, due to the fact that Mg is an intrinsically poor cathode owing to a low exchange current density for HER, alloying additions and impurity elements generally increase cathodic reaction and cathodic activation, resulting in a higher corrosion rates^19^,^20^,^21^,^22^,^23^. Hence, improving the corrosion resistance of Mg via metallurgical alloying routes remains an issue in need of significant work. Nonetheless, a recent breakthrough was demonstrated via the use of metallic arsenic (As) as cathodic poison to supress cathodic reaction and cathodic activation of Mg. In that work, As was metallurgically alloyed with Mg resulting in a significant reduction in the corrosion rate of Mg^24^. On the basis of the demonstrated performance of the Mg-As alloy system, pathways for the potential discovery and development of Mg-alloys with reduced cathodic kinetic and improved corrosion resistance are sought to be pursued. Undoubtedly, the wider use of As as an alloying element is problematic given that As is toxic and potentially unsafe in the handling and melt-based production of Mg-As alloys. Therefore, imparting corrosion protection to Mg in a manner similar to that offered by the Mg-As system, however with less toxic alloying elements, would yield a more practical corrosion prevention strategy for wider implementation in Mg alloys.

The mechanism of cathodic poisoning as offered by As is based on the well document ability of As to serve as a hydrogen recombination poison, and in fact was well validated for the case of Mg corrosion protection when added to a corrosive electrolyte in soluble form^25^. Cathodic poisons are also usually remarkably effective at hindering HER at low concentrations, and characterised by an ability to decrease cathodic current density at a given over-potential^25^,^26^,^27^. Considering such mechanistic factors, the pursuit of an element with similar chemical and electrochemical characteristics as As, however remaining non-toxic, was determined to possibly be germanium (Ge)^28^,^29^,^30^.

In seeking to determine if Ge has been previously alloyed with Mg, there exists an early report by McDonald from 1942^31^, where the mechanical properties of a series of binary Mg alloys with 0.1 to 10 wt.% of Ge addition were investigated, with the 0.5~5 wt.% range of Ge noted as optimum range to improve the yield and tensile strength. More recently, in an isolated study, Kim and co-workers reported Ge containing Mg binary alloys with 0.5~2 wt.% Ge^32^. In that work, the authors did mention that – from limited tests – the corrosion performance of the Ge containing alloy in 3.5% NaCl appeared to be improved relative to pure Mg. However, in that study it was hypothesised that Ge additions refined the alloy grain structure and contributed to surface film stability which resulted in imparting corrosion resistance^32^. However, that hypothesis was not tested, and it is questionable if grain structure plays a significant role in corrosion of heavily alloyed Mg-alloys^33^ and further debatable that Ge can have any role in improved surface films upon the Mg surface^19^,^20^. As such, there is both a paucity of work, and a lack of understanding on the role of alloyed Ge with regards to the corrosion of Mg, and more specifically, no work to date on the influence of Ge on the catalytic activity of Mg towards the HER.

The aim of the present study is to comprehensively study the influence of Ge micro-alloying additions upon Mg, studying the electrochemical and corrosion properties, with the view of the possibility of alloyed Ge serving as a functional non-toxic alloying addition to improve the corrosion resistance of Mg.

## Results

### Physical characterisation of Mg-Ge alloys

**Figure 1:**
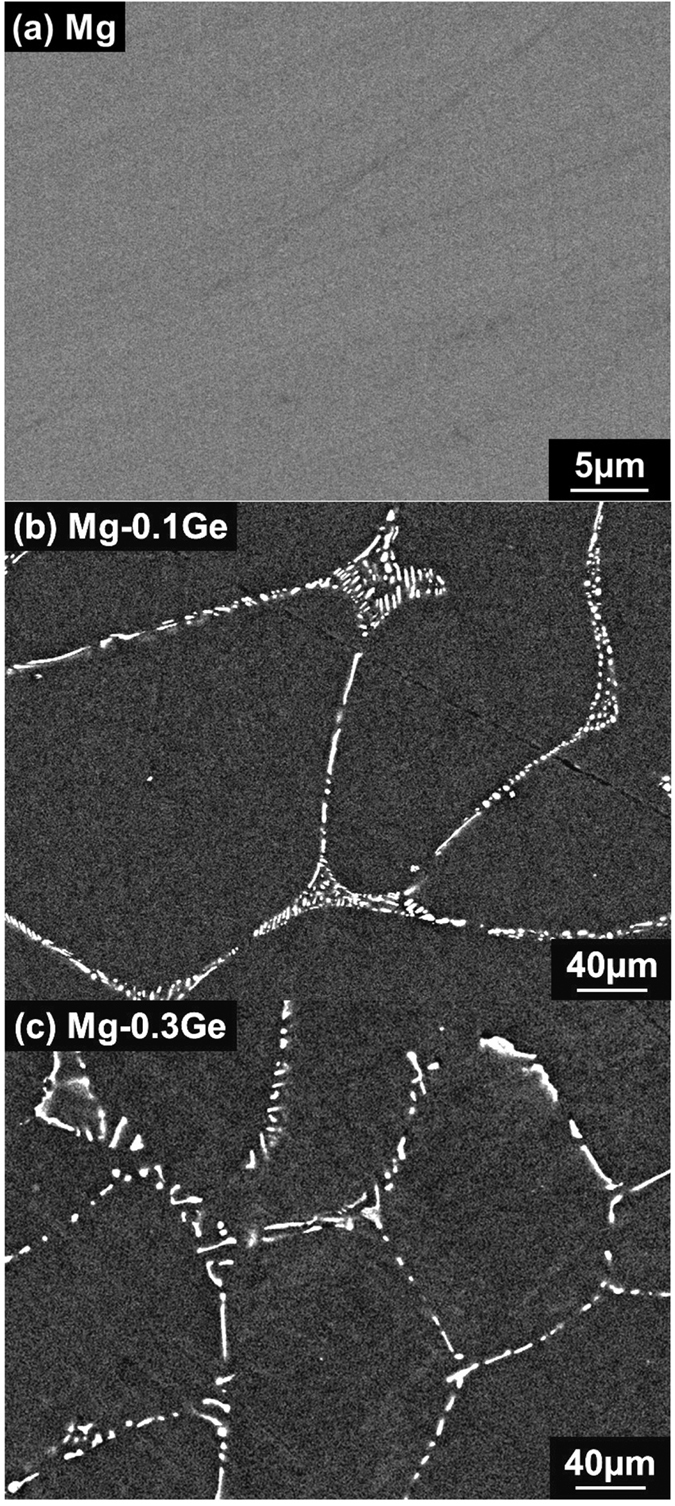
Scanning electron micrographs in backscattered electron mode for (**a**) pure Mg, (**b**) Mg-0.1Ge and (**c**) Mg-0.3Ge.

The microstructure of pure Mg and the two custom produced Mg-Ge alloys as determined from SEM using backscattered electron (BSE) imaging are shown in Fig. 1.

We observe that pure Mg is single phase and free of intermetallics (Fig. 1a), however the Mg-Ge alloys include the presence of a second phase, as evidenced by the bright contrast in Fig. 1b,c. The bright contrast in BSE mode is indicative of higher atomic number elements (in this case Ge). The presence of such a second phase was expected on the basis that the reported solid solubility of Ge in Mg is low (a maximum of 0.01 wt.% Ge can be dissolved in Mg at 602 °C^34^). The microstructure of the Mg-Ge alloys can be described as lamellar eutectic mixtures of “rod shape like” intermetallics present along grain boundaries. The stoichiometry of the observed intermetallic is confidently ascribed to Mg_2_Ge, based on it being the only possible phase from the equilibrium phase diagram over a wide range of Ge additions^34^, but also confirmed by energy dispersive x-ray spectroscopy (not reported herein as the stoichiometry is not controversial).

**Figure 2:**
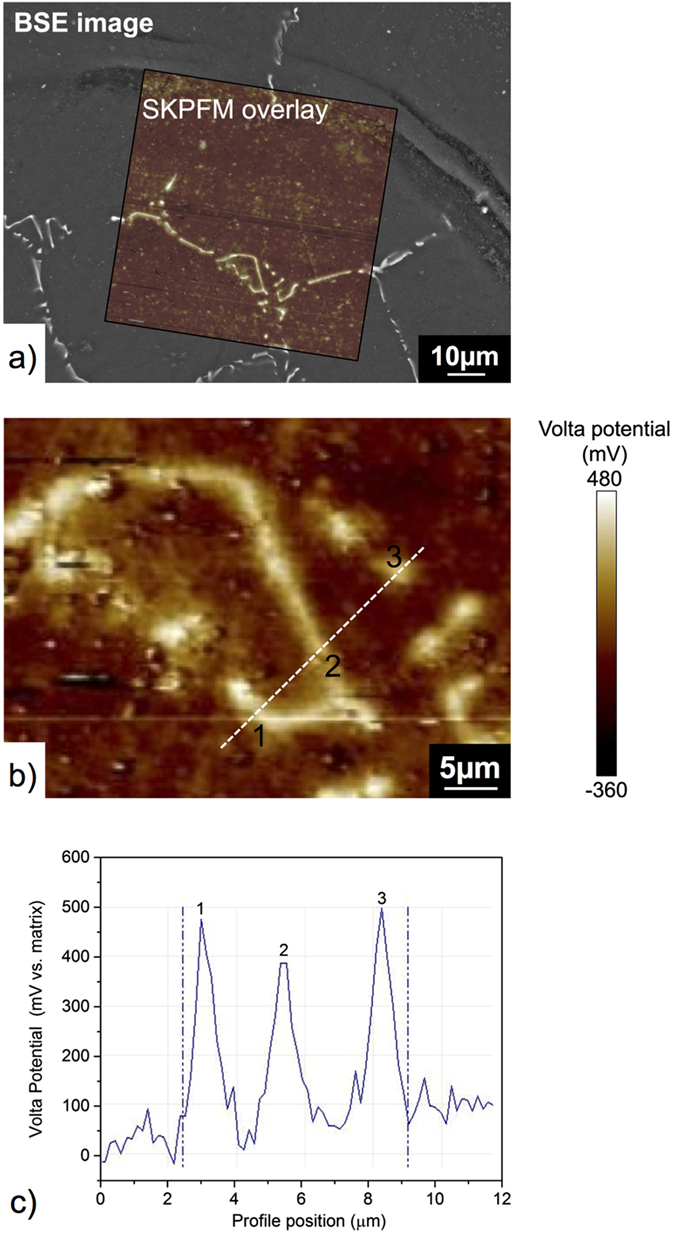
(**a**) Overlaid BSE image and SKPFM image, (**b**) Volta potential map of Mg-0.3Ge alloy showing the relative potential of the Mg_2_Ge second phase and matrix and (**c**) Line scan data for the Volta potential corresponding to the dashed line region in (**b**).

In order to further assess the microstructure of the Mg-Ge alloy, the results of Volta potential measurements upon Mg-0.3Ge, along with corresponding BSE images are presented in Fig. 2.

It is observed that in conjunction with the compositional difference as confirmed by BSE imaging, there also exists a difference in the measured Volta potential – measured by the SKPFM (scanning Kelvin probe force microscopy) method upon microstructural features (Fig. 2a).

As depicted clearly in Fig. 2b, the bright regions of Volta potential correspond to Mg_2_Ge which presents a notably higher Volta potential than the alloy matrix. A quantitative representation of the Volta potential profile for Mg-0.3Ge alloy corresponding to the line overlay in Fig. 2b is reported in Fig. 2c. The bright points from the Volta potential map corresponded Mg_2_Ge, which presents a Volta potential value ~400 mV higher than the matrix phase (where the methodology employed here, and a general overview of the determination and analysis of Volta potentials is reported in the previous work^35^). The increased Volta potential in the case of intermetallics which populate Mg, is suggestive that intermetallic second phase may serve as a localised cathode upon micro-galvanic coupling with the surrounding matrix anodes – bearing in mind the caveat that the Volta potential is not measured in a corrosive electrolyte, but in open dry air (laboratory) environment.

### Potentiodynamic polarisation response of Mg-Ge alloys

**Figure 3:**
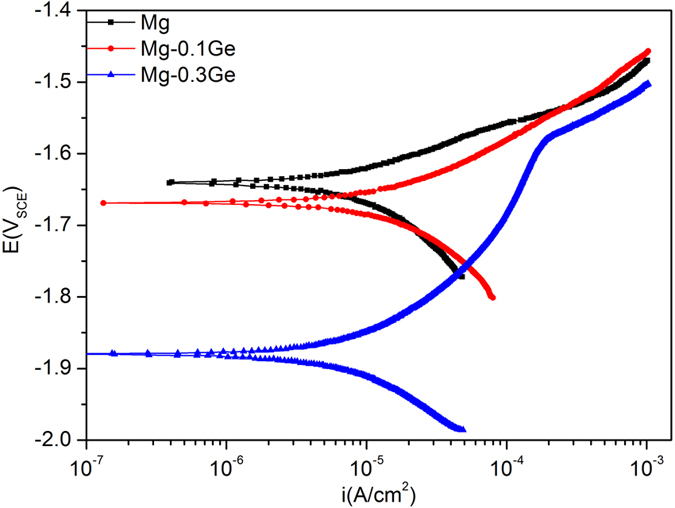
Potentiodynamic polarisation data in 0.1 M NaCl for pure Mg, Mg-0.1Ge, and Mg-0.3Ge.

The typical potentiodynamic polarisation response of specimens tested is presented in Fig. 3.

The Mg-0.1Ge alloy studied herein presents rather similar anodic kinetics to pure Mg, which indicates that the 0.1Ge alloy loading had a minor effect on the measured anodic kinetics. Similarly, the 0.1Ge alloy loading also showed a minor difference in the cathodic kinetics as seen from the potential range in Fig 3, however the unique cathodic kinetics from cathodic polarisation are reported further below.

**Figure 4:**
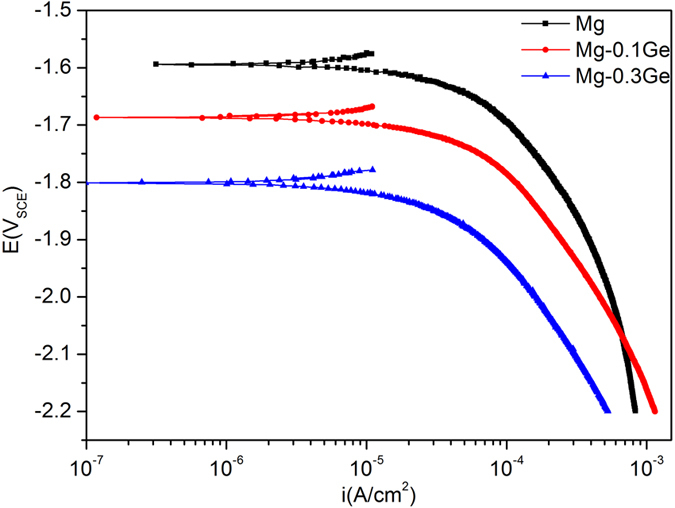
Cathodic potentiodynamic polarisation data in 0.1 M NaCl for pure Mg, Mg-0.1Ge and Mg-0.3Ge.

In the case of the 0.3Ge alloy loading, we observed a marked change in the electrochemically-measured kinetics. Perhaps most striking is the significantly lower E_corr_ value for Mg-0.3Ge (presenting an E_corr_ of ~−1.88 V_SCE_), which is then able to reveal an apparent ‘passive window’ for the alloy, between the range of E_corr_ and ~−1.6 V_SCE_. At higher overpotentials, the anodic dissolution kinetics are similar for Mg and the Mg-Ge alloys. The response of the Mg-0.3Ge alloy is principally dictated by an alteration in cathodic kinetics; however, the high passive current density revealed for Mg-0.3Ge also contributes to the realisation of a lowered E_corr_. In order to have a fuller comparison of cathodic kinetics between the studied alloys, unique cathodic polarisation tests commencing from the corrosion potential and scanned to increasingly negative potentials are reported in Fig. 4.

From the cathodic scans (Fig. 4), we observe a distinct trend of cathodic current density decreasing with increasing Ge addition, the effect being minor for 0.1Ge, but considerable for 0.3Ge. The cathodic scans indeed reveals the ability of Ge addition to suppress the cathodic reaction upon Mg over a range of potentials. Whilst it may be obvious, we make the point here that the decreased E_corr_ of Mg-Ge alloys corresponds to improved corrosion resistance, as the decreased E_corr_ corresponds to the reduction of cathodic kinetics.

### Hydrogen evolution and mass loss of Mg-Ge alloys immersed in 0.1M NaCl

**Figure 5:**
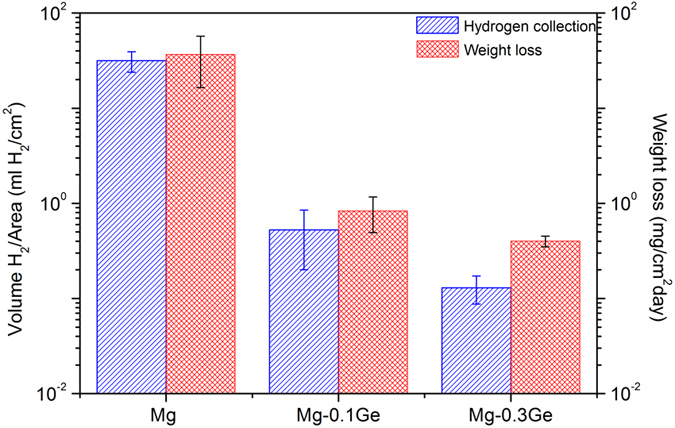
Hydrogen collection and mass loss results of pure Mg, Mg-0.1Ge and Mg-0.3Ge following 24 h immersion at OCP in 0.1 M NaCl.

Whilst potentiodynamic polarisation allows quantification of electrochemical kinetic information regarding Mg corrosion (namely the rates of the partial corrosion reactions) a measure of corrosion rates at open circuit is independently useful. Recent published work by *King et al.* suggested a combined technique including electrochemical impedance spectroscopy (EIS), mass loss and hydrogen for an accurate determination of long term or ongoing rates of Mg corrosion^36^, which was validated by Bland^18^ for Mg alloys. Herein, we do not perform EIS, but adopted simultaneous hydrogen collection and mass loss testing for a duration of 24 h in 0.1 M NaCl, the results of which are shown in Fig. 5.

Once again, the Mg-Ge alloys are benchmarked against pure Mg, and it is revealed that both Mg-Ge alloys tested revealed an appreciable reduction in the rate of corrosion relative to Mg. In fact, the reduction in corrosion rate was at least one order of magnitude judged by both the mass lost (in 24 h) and hydrogen evolved, which is remarkable. What we observe from Fig. 5, which may not have been anticipated from the potentiodynamic polarisation results alone, is that the 0.1Ge loading was also very potent at reducing corrosion. This phenomenon, as described below, is most likely not captured by the potentiodynamic testing on the basis that the beneficial effect is likely from the ability to supress cathodic activation (where the cathodic activation is a time dependent phenomenon).

It is noted that further alloying Mg with 0.3Ge to is even more beneficial for improvement of corrosion resistance, particular in the suppression of hydrogen evolved. The Mg-0.3Ge alloy evolved less than 0.2 ml of H_2_/cm^2^/day, which is comparatively insignificant when contrast to reports of Mg corrosion in the open literature.

### Corrosion morphology

**Figure 6:**
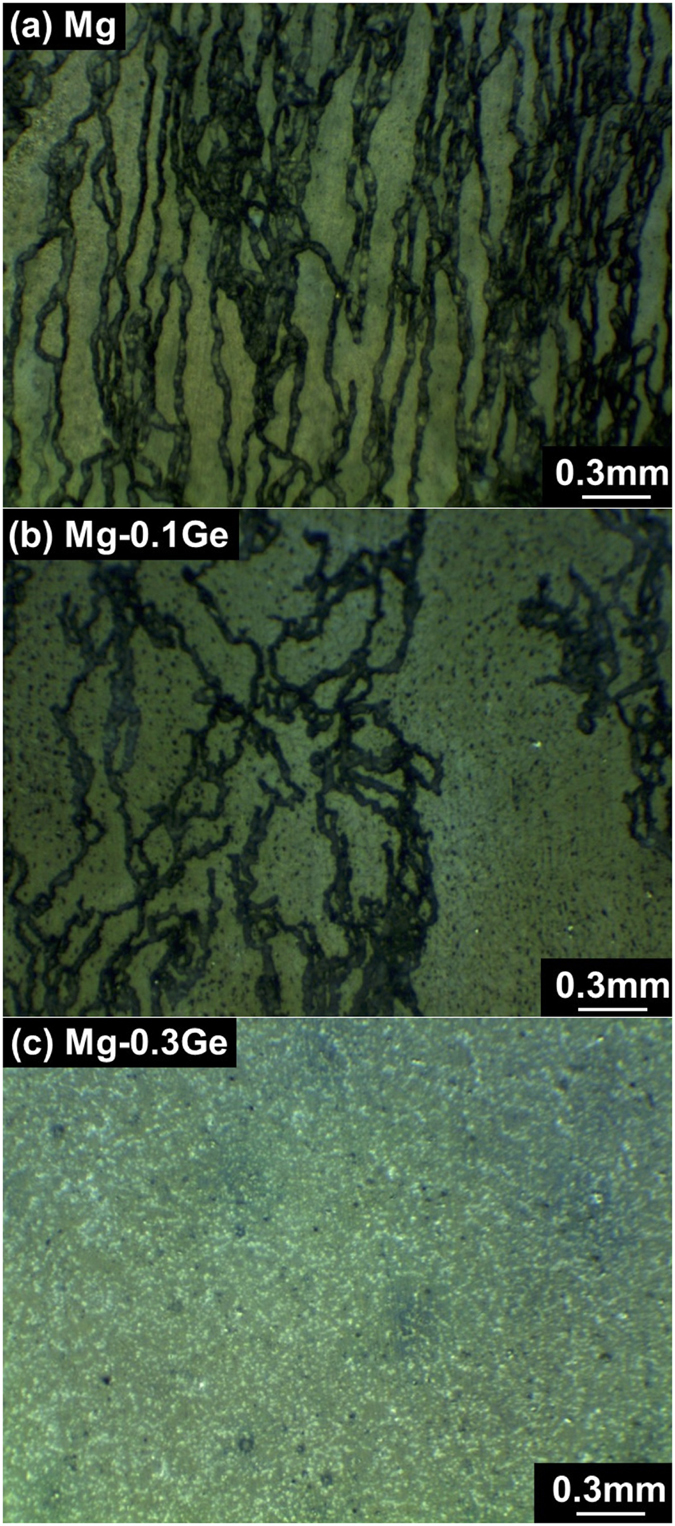
Optical micrographs of corrosion morphology of (**a**) Mg, (**b**) Mg-0.1Ge and (**c**) Mg-0.3Ge following 24 h immersion in 0.1 M NaCl.

The optical micrographs of specimen surfaces following corrosion exposure (i.e., the mass loss and hydrogen collection tests which were conducted for 24 h in 0.1 M NaCl), are presented in Fig. 6.

The corrosion morphology observed presents a typical “filiform like” dark corrosion morphology that is uniformly distributed over the whole exposed surface for the case of pure Mg (Fig. 6a). Such “filiform like” corrosion morphology was associated with the spreading/propagation of corrosion where local anode and cathode sites dynamically evolve. Following corrosion evolution, there is evidence to support that the “filiform like” dark areas observed are associated with regions of enhanced cathodic kinetics arising from prior anodic polarisation, based on spatial and temporal studies using the scanning vibrating electrode technique (SVET) method^17^.

In the starkest of contrast to pure Mg, the corrosion morphology upon the Mg-0.3Ge alloy was very minimal, and completely superficial, whilst devoid of filiform-like corrosion and dark regions. In fact, the discrete superficial corrosion morphology following exposure to 0.1 M NaCl is similar to that observed in previous work which studied microalloying additions of As^24^. The corrosion morphology for the Mg-0.3Ge alloy reveals that Ge alloying effectively hinders the propagation of filiform-like attack. This function, not only resulted in a reduced rate of corrosion, however if the presence of the dark film is indeed indicative of cathodic activation, then the corrosion morphology is indeed indicative of reduced anodically enhanced cathodic activation.

### Galvanostatic-potentiostatic response of Mg-Ge alloys

**Figure 7:**
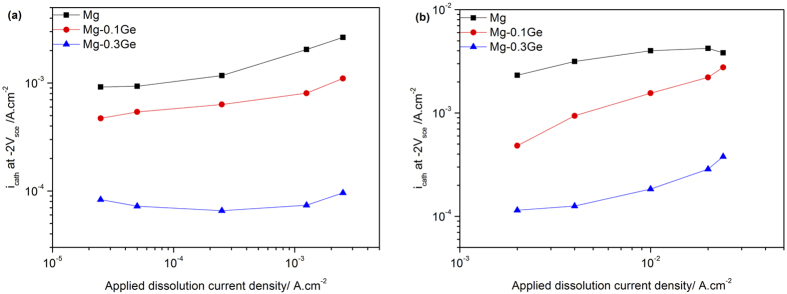
Abridged results of measured cathodic current density at −2.0 V_SCE_ in response to prior anodic current signal of (**a**) (0.025 to 2.5 mA/cm^2^) and (**b**) (2 to 24 mA/cm^2^).

Cyclic galvanostatic-potentiostatic tests employing a custom electrochemical signal were carried out to further assess the effect of Ge additions on the electrochemically measured cathodic activation in response to anodic polarisation. Such tests were designed to provide a stepwise increasing galvanostatic anodic polarisation to the specimens, and each step was interrupted by a potentiostatic cathodic polarisation at the fixed value of −2 V_SCE_. This means that the resultant cathodic current could be compared at the same potential following a period of increasing dissolution. The results of such tests are abridged into Fig. 7.

The results of the specimens studied for exposure regimes that range in net anodic dissolution rates from 0.025 to 2.5 mA/cm^2^ (which correspond to the galvanostatic steps that are separated by a potentiostatic hold at −2.0 V_SCE_) are summarised in Fig. 7a. The raw data from which such analysis occurred has also been provided as supplementary information.

In the case of pure Mg, there is evidence that there is an increasing cathodic current density that rises with increasing prior applied anodic current density (Fig. 7a). The notion of enhanced catalytic activity of Mg in response to prior anodic polarisation whether cyclic or otherwise is in line with the previous work which provided electrochemical evidence for cathodic activation on cyclic anodic polarised Mg surface^37^. In contrast, the measured cathodic current density (across a range of prior anodic current exposures) was reduced with increasing alloy concentration of Ge. For the case of Mg-0.3Ge, a one order magnitude lower cathodic current density than pure Mg was recorded – and this was irrespective of prior anodic exposure. Furthermore, a relatively constant value of the measured cathodic current density upon Mg-0.3Ge suggests that the alloy remains more resistant to cathodic activation, indicating the ability of alloyed Ge addition to hinder such cathodic activation. This reliability and ‘stability’ is considered very promising in the context of Mg-anode performance in primary Mg batteries.

Galvanostatic-potentiostatic cycling was further carried out, the results of which are seen in Fig. 7b, for conditions of higher applied anodic current densities (from 2~24 mA/cm^2^). A similar trend was observed for pure Mg, and with lower cathodic currents realised with Ge additions. However, what is noted is that a slight cathodic activation of Mg-0.3Ge was observed for prior applied anodic currents of >10 mA/cm^2^ (which corresponds to a very high rate of prior anodic exposure – and significantly higher than any free corrosion or even battery applications).

## Discussion

Custom binary Mg-alloys with micro alloying additions of Ge were studied using electrochemical and corrosion testing, coupled with microstructural characterisation. It was revealed by complementary tests that low level Ge additions significantly reduced the cathodic kinetics (i.e. the HER) upon Mg, and the impact was (i) reduced corrosion rates from mass loss testing, (ii) reduced hydrogen evolved during corrosion, and (iii) a drastic alteration of the morphology typical of corrosion for specimens immersed in 0.1 M NaCl. All of these phenomena are self-consistent, and were ascribed to the ability of Ge to ‘poison’ the HER, which was also demonstrated herein to occur, as evidenced via cyclic electrochemical testing.

We report that the use Ge additions to Mg, were capable of achieving a similar outcome or performance with the previously Mg-As system^24^. However in the present study we also performed cyclic electrochemical testing – which further asserts the possibilities of Ge additions to provide a safe and practical strategy for more corrosion resistant Mg alloys. To this end, significant future work awaits, with respect to at least three areas, namely (i) imparting the Ge-effect to more complex Mg-alloys which have more substantial and intentional alloying and higher strengths than pure Mg, and (ii) testing of the Ge-effect in Mg electrodes which are nominally prone to excessive HER and “parasitic discharge” occurring from the during charge/discharge cycling^6^,^8^,^12^. Parasitic discharge is in itself a manifestation or result of cathodic activation^37^, and that is the same cathodic activation that Ge is able to stifle, (iii) any unconsidered deleterious effect that may result from possible accumulation of H_abs_ if hydrogen is unable to be recombined.

**Figure 8:**
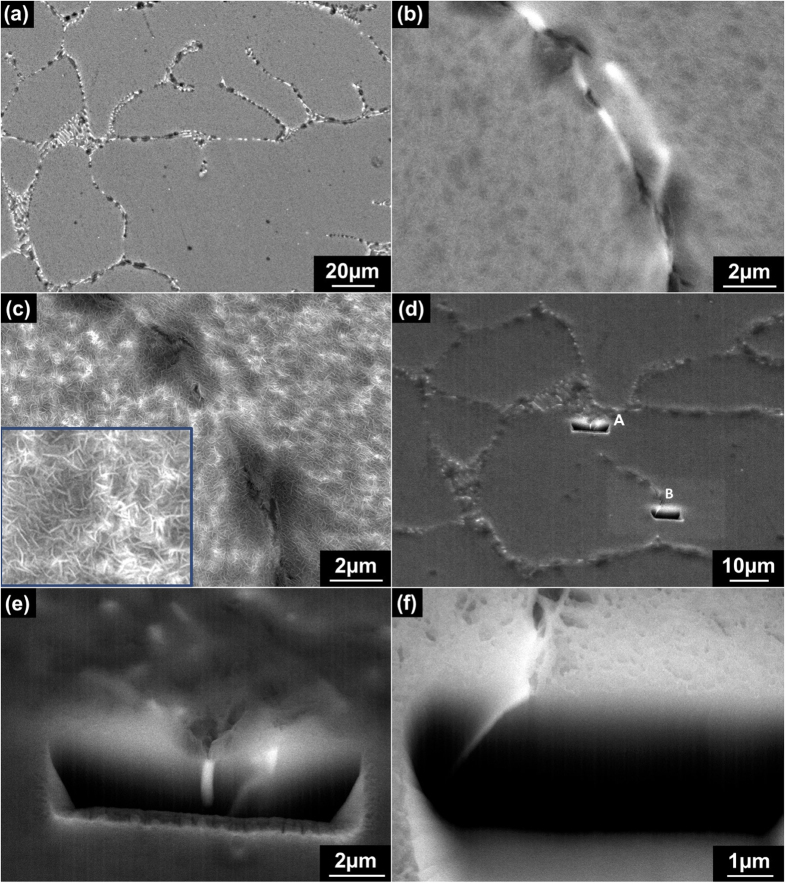
SEM images of corroded Mg-0.3Ge alloy following 1 h immersion of 0.1 M NaCl solution: (**a**) low magnification (BSE); (**b**) high magnification (BSE); (**c**) high magnification (SE); (**d**) SEM image (BSE) showing the as marked site A and site B for the imaging of cross-section via FIB milling; (**e**) SEM image (BSE) of cross-section of site A; (**f**) SEM image of cross-section of site B.

In an attempt to rationalise the results reported to now, further microscopy was carried out upon Mg-0.3Ge following 1 h immersion in 0.1 M NaCl. This analysis was done in order to more closely study the early stages of corrosion morphology for physical clues as to the electrochemical assertions, with such corresponding electron micrographs shown in Fig. 8.

From SEM analysis, no typical filiform-like pattern of corrosion was seen, and only discrete corrosion sites were observed upon the alloy surface (Fig. 8a). From Fig. 8a, and then from its higher magnification variant (Fig. 8b), it is apparent the extent of corrosion was minimal relative to an Mg-alloys reported to date, whilst the corrosion morphology upon Mg-0.3Ge was clearly different from the conventional morphology observed on Mg and Mg-alloys, which is nominally typified by extensive corrosion of the matrix^19^. Instead, as noted from Fig. 8a,b (both BSE images) the discrete sites of corrosion are near to, the interface of the Mg_2_Ge (bright) phase. Figure 8c is the partner image to Fig. 8b, however presents secondary electron (topographical) contrast. We observe two distinct features, in that the corrosion damage can be readily observed discrete sites, whilst the matrix phase is covered by a uniform distribution of a dense “needle-like” hydroxide film, which uniformly covers the whole surface of the alloy. Two sites that correspond to the vicinity of discrete corrosion were selected for the characterisation of cross sections of corrosion valleys, via FIB milling (the sites are depicted in Fig. 8d).

As seen in Fig. 8e, corresponding to corrosion site A, the cross section of the so-called corrosion valley reveals shallow corrosion damage, observed to be less than ~1 μm in depth. This indicated that the dissolution which occurred was minimal.

FIB milling (in both Fig. 8e,f) revealed that second phase intermetallic particles, Mg_2_Ge, were in the vicinity of corrosion valleys. Recalling the results section, it is believed that Mg_2_Ge will serve the role of local cathode. This assertion was qualified on the basis that the SKPFM results suggest that Mg_2_Ge will be a favourable cathode site if the measured relative nobility is valid in the case of 0.1 M NaCl aqueous solution. Secondly, the corrosion morphology showed that matrix phase was almost intact and ‘activated’ sites were not really observed. However, the overall corrosion morphology observed in Fig. 8 has no precedent in the study of Mg-corrosion to date. For example, if the Mg_2_Ge is a preferred cathode, then the alkalinity generated at the cathode should ‘push’ the corrosion sites away from the cathode, whereas in this instance they seem to be localised adjacent to the cathode. Furthermore, given the tendency for Mg (matrix) to support cathodic reactions as now well-known^38^, the lack of any ‘activated’ sites on the matrix is suggestive that either all cathode activity is away from the matrix, or that the alloy potential during corrosion (when alloyed with Mg) supresses the matrix cathodic activity. This is indeed an aspect of work that warrants detailed further study, via *in situ* methods such as SVET and SECM.

Nonetheless, and in spite of the need for closer analysis, the information in the present study reveals the potentially significant benefits of alloying Mg with Ge. Some points which can be determined from the electrochemical and corrosion analysis and the microscopy as presented in Fig. 8, are summarised below in the context of high level phenomenology regarding the mechanistic role of Ge additions.

1. Ge additions will induce the presence Mg_2_Ge, when Ge is added above the solubility limit; the solubility limit of Ge in Mg being low (0.01 wt.%).
2. Mg_2_Ge is likely to serve as a local cathode at the potential of Mg-Ge alloys, based on the premise of SKPFM. Unique electrochemical testing on Mg_2_Ge appears to be essential future work, given the promise the system presents. In spite of Mg_2_Ge phase serving as a local preferred cathode site, the corresponding anodic current appears to only translate to what is superficial damage.
3. If the relative nobility of Mg_2_Ge persists in aqueous environments, then at that the alloy potential, Mg_2_Ge is likely the persistent and favoured cathode, which may limit the ability of the matrix to serve in the role of cathode (and potentially restrict cathodic activation). Whilst the mechanism of cathodic activation requires further work - more generally in the Mg field - several recent studies^39^,^40^,^41^ showed that the development of a hydroxide film Mg(OH)_2_ film on the surface of Mg is a key factor in cathodic activation. The role of Ge alloying in terms of its influence on MgOH_2_ growth requires focused work.

Based on the results herein, it is reasonable to assert that Ge alloying additions beneficially alter the corrosion rate, corrosion propagation, corrosion morphology, and hydrogen evolution, upon Mg.

## Conclusions

In the present work, the influence of microalloying of high purity Mg (less than 100 ppmw Fe) with Ge was assessed in the context of corrosion control, and the following conclusions can be drawn.

1. Metallurgical alloying of Mg with the non-toxic element Ge, resulted in significantly reduced cathodic kinetics for Mg.
2. Intermetallic second phase particles within Mg-Ge alloys, namely Mg_2_Ge, were determined to be more noble than the Mg-matrix phase (by a Volta potential difference of ~400 mV) as determined SKPFM. It is posited that this relationship (i.e. Mg_2_Ge serving as a cathode phase) is retained in aqueous environments, and such Mg_2_Ge sites are preferred sites of reduction. It is posited that such Mg_2_Ge cathode sites are concomitantly ‘sluggish’ sites of reduction. However, the mode and extent to which such cathode sites supress the cathodic activation of the Mg matrix as judged by corrosion morphology is complex and will require future work. A complete understanding of the effect of alloyed Ge with regards to electrochemistry will require
3. The post exposure corrosion morphology of Mg was altered from widespread “filiform like” morphology in 0.1 M NaCl, to minor discrete corrosion sites with Ge alloying. The corrosion sites upon Mg-Ge alloys were associated with the vicinity of intermetallic particles as validated by cross sectional microscopy.
4. A survey of mass loss, hydrogen evolved and electrochemical testing indicated Ge additions imparted a significant corrosion resistance to Mg, in addition to some resistance to cathodic activation. It is appreciated that full quantification of the latter requires further work, both over longer term cycling and to assess any second order effects that may occur (such as the fate of stymied hydrogen recombination).
5. The suppression of the cathodic activation of Mg has significant ramifications for Mg electrodes in cathodic protection or primary battery systems.

## Methods

### Materials

Alloys were prepared from pure Mg (Amac, Australia) and pure Ge (Alfa-Aesar, USA). Alloy preparation involved melting via a Leybold-Heraeus ISO1/III induction furnace. Prior to melting, the induction furnace chamber was evacuated and backfilled with protective gas, AM-cover (10 wt.% of tetrafluoroethane and 90 wt.% argon) to a positive pressure (maintained slightly above 1 atm). The alloy melt was heated to 750 °C in a graphite coated steel crucible. The melt was then held for 30 min during which it was stirred at least 4 times to ensure mixing. The melt was finally cast into a graphite coated rectangular steel mould preheated to 200 °C, and left to cool.

In addition the pure Mg, two Mg-Ge alloys with a target alloying content of 0.1 wt.% and 0.3 wt.% Ge, were successfully cast in this work, and the precise composition of which was independently analysed (Spectrometer Services, Australia) by inductively coupled plasma atomic emission spectroscopy (ICP-AES) and reported in Table 1. Trace analysis indicated that for all samples, impurity levels such as iron (Fe), were below the so-called “impurity threshold”^19^,^42^.

### Electrochemical tests

Specimens for electrochemical tests were cut from the cast ingots metallographically prepared to 2000 grit SiC paper finish under ethanol. A conventional three electrode electrochemical configuration was employed using a flat cell (Princeton Applied Research, USA) with a 1 cm^2^ working electrode area exposed to quiescent 0.1 M NaCl (pH = 6, open to air) with 0.95 atm hydrogen gas pressure at 25 °C. A saturated calomel electrode (SCE) reference electrode and Pt-mesh counter electrode were used, and tests were carried out using VMP 3Z potentiostat (Bio-logic, USA).

To reveal the effects of alloying on the anodic and cathodic kinetics of alloys studied, potentiodynamic polarisation testing was conducted. Prior to testing, samples were conditioned at the open circuit potential (OCP) for 10 min to ascertain a relatively stable potential. Polarisation was carried out at scan rate of 1 mV/s. Two potentiodynamic tests were employed in unique isolated experiments. This included an upward scan from an initial potential 200 mV below OCP for determination of overall anodic kinetics, and in another set of experiments, a downward scan from 20 mV more positive than OCP to a potential of −2.2 V versus SCE for assessment of cathodic reaction kinetics. Each tests was reproduced at least five times and a representative scan is shown.

To evaluate the catalytic reactivity of Mg samples towards HER in response to prior anodic dissolution, a custom combination of galvanostatic and potentiostatic cyclic testing was applied as per prior works^37^. The cyclic testing was executed using a stepwise increasing current from 0.025 to 2.5 mA/cm^2^ each step of which was applied for 2 min and separated by a fixed potentiostatic signal of −2 V_SCE_ (applied for 1 min) to assess the cathodic reaction kinetics as a function to the prior anodic galvanostatic polarisation. This cyclic test was also repeated using a regime of higher anodic current density (from 2~24 mA/cm^2^) to provide a comprehensive assessment of the prevalence of cathodic activation.

### Physical characterisation

The microstructure of as-cast samples were analysed using a JEOL 7001F scanning electron microscope (SEM) in back-scattered electron mode (BSE). Specimens for SEM analysis were metallographically prepared to a 0.05 μm surface finish using colloidal suspension. In addition, an FEI Quanta 3D-FEG dual beam scanning electron microscope and focused ion beam (FIB) was also employed for the imaging of samples following 1h exposure to 0.1 M NaCl. Some site specific FIB milling was employed to cross section apparent sites of corrosion and followed by imaging in both secondary electron (SE) and BSE mode.

The surface corrosion morphology of specimens following 24 h immersion in 0.1 M NaCl were characterised by an Olympus upright optical stereo microscope.

The Volta potential measurement was performed using atomic force microscopy (AFM) imaging coupled with scanning Kevin probe force microscopy (SKPFM). Testing was carried out in open dry air condition at room temperature (25 °C). Prior to SKPFM, metallographically prepared samples were finalised with the following preparation methodology. Polishing with non-aqueous diamond slurries to a 1 μm finish, and an ultimate polish with a 0.01 μm non-aqueous alumina slurry lubricated with ethanol. Alumina slurry was utilised in the final step to avoid galvanic interaction between silica-based polishing compounds, and samples were cleaned by ultrasonicating in pure ethanol. Prior to imaging, samples were cleaned with lens tissue and HPLC/spectrophotometric grade ethanol. An electrical connection between the sample surface and the AFM stage was made with silver paste and verified with a handheld voltmeter prior to testing. Imaging was carried out with a Bruker Dimension Icon AFM operating in FM (frequency modulation) PF-KPFM mode. The PF in PF-KPFM indicates the manufacturer’s proprietary PeakForce tapping mode was employed for topographical imaging. Topographical imaging is not shown herein, however subsequent to the acquisition of each individual line in the topographical scan, SKPFM measurements were conducted in lift mode using frequency modulation based detection and nullification of the tip-sample surface potential. Bruker PFQNE-AL probes (5 nm radius of curvature) were selected to provide optimal Volta potential resolution. The aluminium coating on the backside of the SiN_3_ PFQNE-AL probes served as the reference in the SKPFM measurements and enabled quantitative determination of absolute surface potentials via determination of the work function relative to pure metal standards in open dry air (laboratory) environment.

### Hydrogen collection and mass loss

Preparation and cleaning of corrosion test specimens was carried out in accordance to the ASTM-G1-03 standard^43^. Immersion test specimens were cut from the Mg alloys castings into cuboids with a ~5 cm^2^ surface area. All specimen surfaces were ground to a 1200 grit SiC finish and sonicated in ethanol, accurately weight and measured prior to mass loss and hydrogen collection testing. An inverted burette and funnel set up in 0.1 M NaCl was applied for the collection of hydrogen during corrosion. Following the exposure period, chromic acid solution (200 g/L chromium trioxide, 10 g/L silver nitrate and 20 g/L barium nitrate) was used to remove surface corrosion products.

## Additional Information

**How to cite this article**: Liu, R. L. *et al*. Controlling the corrosion and cathodic activation of magnesium via microalloying additions of Ge. *Sci. Rep.* **6**, 28747; doi: 10.1038/srep28747 (2016).

## Supplementary Material

Supplementary Information

## Figures and Tables

**Table 1:** Composition of samples tested as independently determined by ICP-AES.

**Wt.%**	**Mg**	**Ge**	**Fe**	**Mn**	**Al**	**Zn**	**Cu**	**Si**	**Ca**
Mg	Bal.	0.000	0.003	0.023	0.004	<0.001	<0.001	<0.04	0.002
Mg-0.1Ge	Bal.	0.088	0.003	0.023	0.004	<0.001	<0.001	<0.04	0.002
Mg-0.3Ge	Bal.	0.330	0.001	0.025	0.01	<0.01	<0.001	<0.02	0.01
