# Supplementary material for: Controlling the corrosion and cathodic activation of magnesium via microalloying additions of Ge
Source: Sci Rep. 2016 Jun 28;6:28747. doi: 10.1038/srep28747 (PMC4923887; doi:10.1038/srep28747)
Supplement: Supplementary Information [file srep28747-s1.doc]

Supplementary information:

**Controlling the corrosion and cathodic activation of magnesium via microalloying additions of Ge**

R.L. Liu^1,*^, M.F. Hurley^2^, A. Kvryan^2^, G. Williams^3^, J.R. Scully^4^ and N. Birbilis^1^

^1^Department of Materials Science and Engineering, Monash University, Clayton, VIC, Australia

^2^College of Engineering, Department of Materials Science and Engineering, Boise State University, Boise, Idaho, USA

^3^Materials Research Centre, College of Engineering, Swansea University, Singleton Park, Swansea, Wales, UK

^4^Department of Materials Science and Engineering, The University of Virginia, Charlottesville, VA, USA


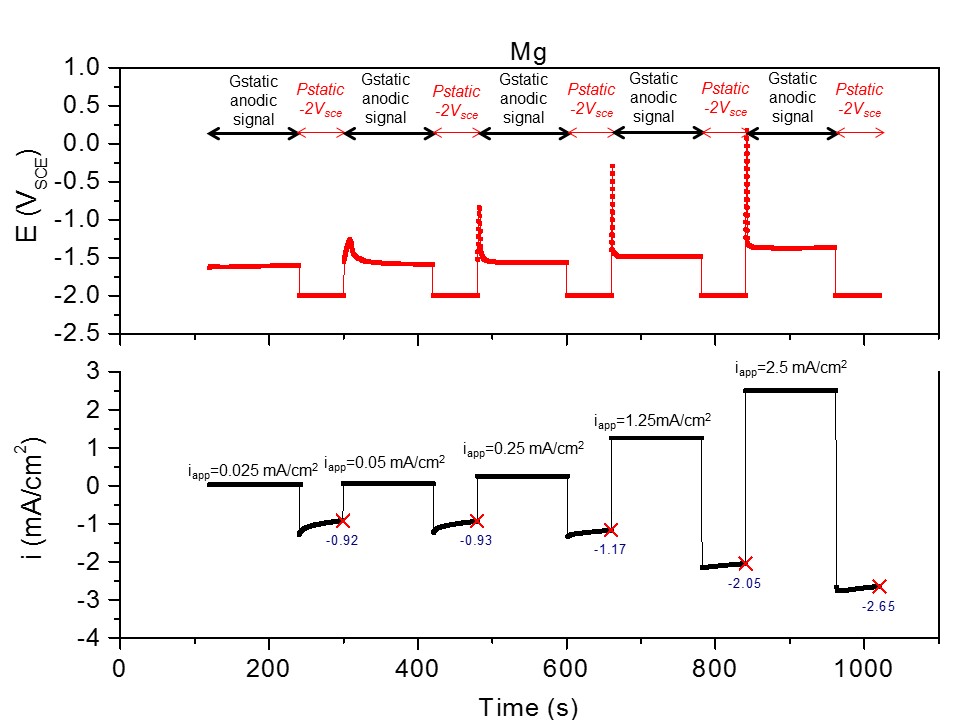
(a)


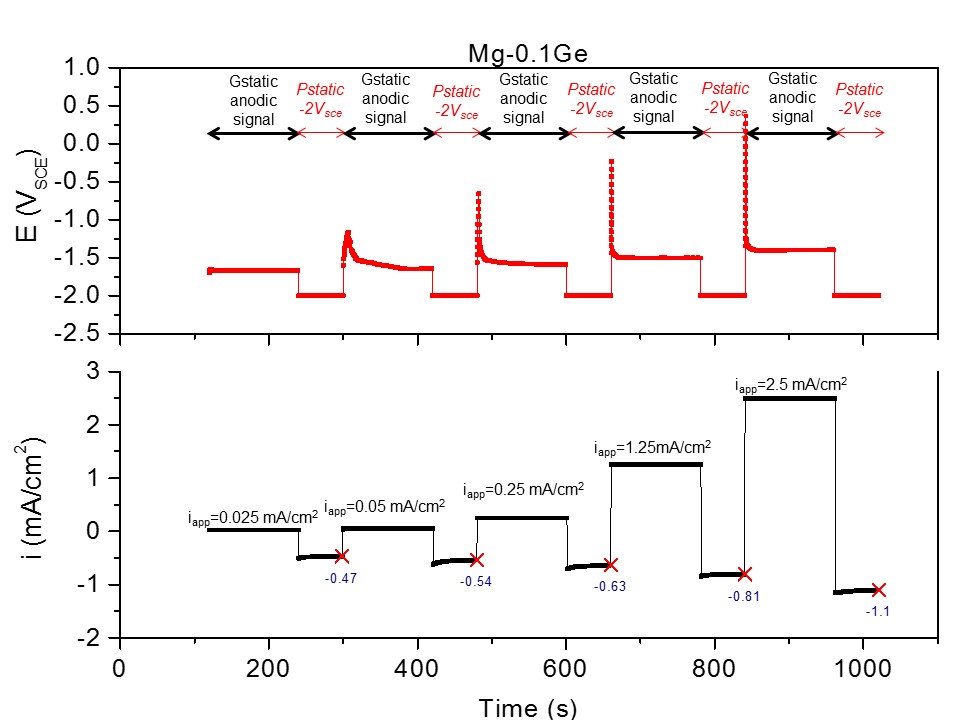
(b)


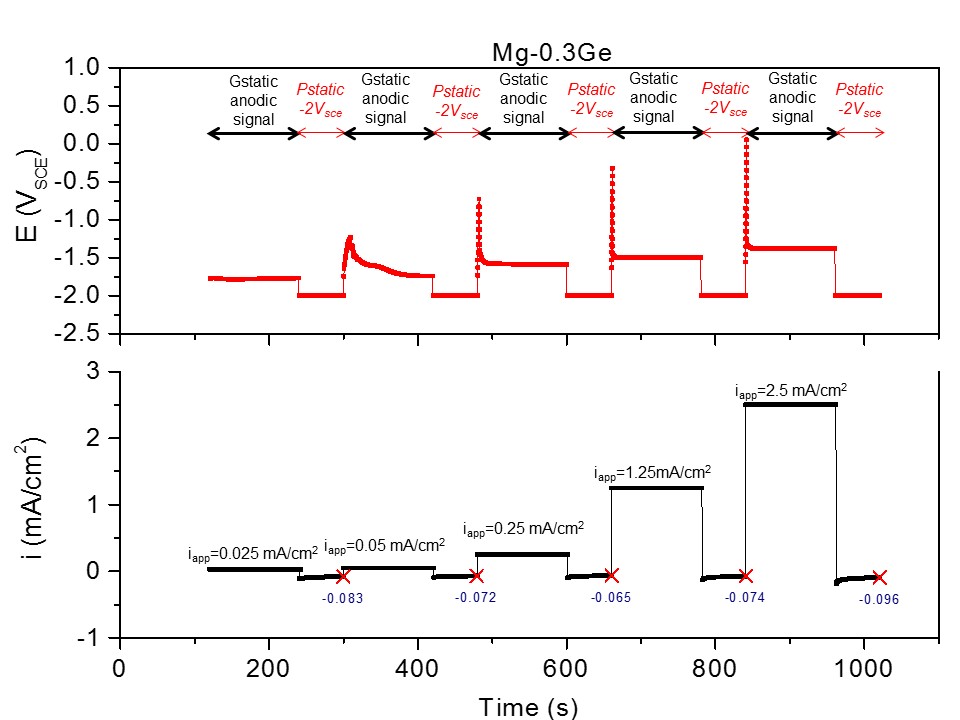
(c)

**Figure S1.** Applied and measured electrochemical currents and potentials from a custom cyclic electrochemical signal for (a) Mg, (b) Mg-0.1Ge and (c) Mg-0.3Ge in 0.1 M NaCl at 25 °C. The applied signal involved an anodic current applied in a stepwise manner from (0.025 to 2.5 mA/cm^2^) with 2 min duration. In between each current step, a 1 minute potentio-static signal at fixed potential of -2 V_SCE_ was applied, and the measured cathodic current (as marked “X” symbol on the curve) was recorded for comparison of cathodic current in response to a prior anodic signal.
